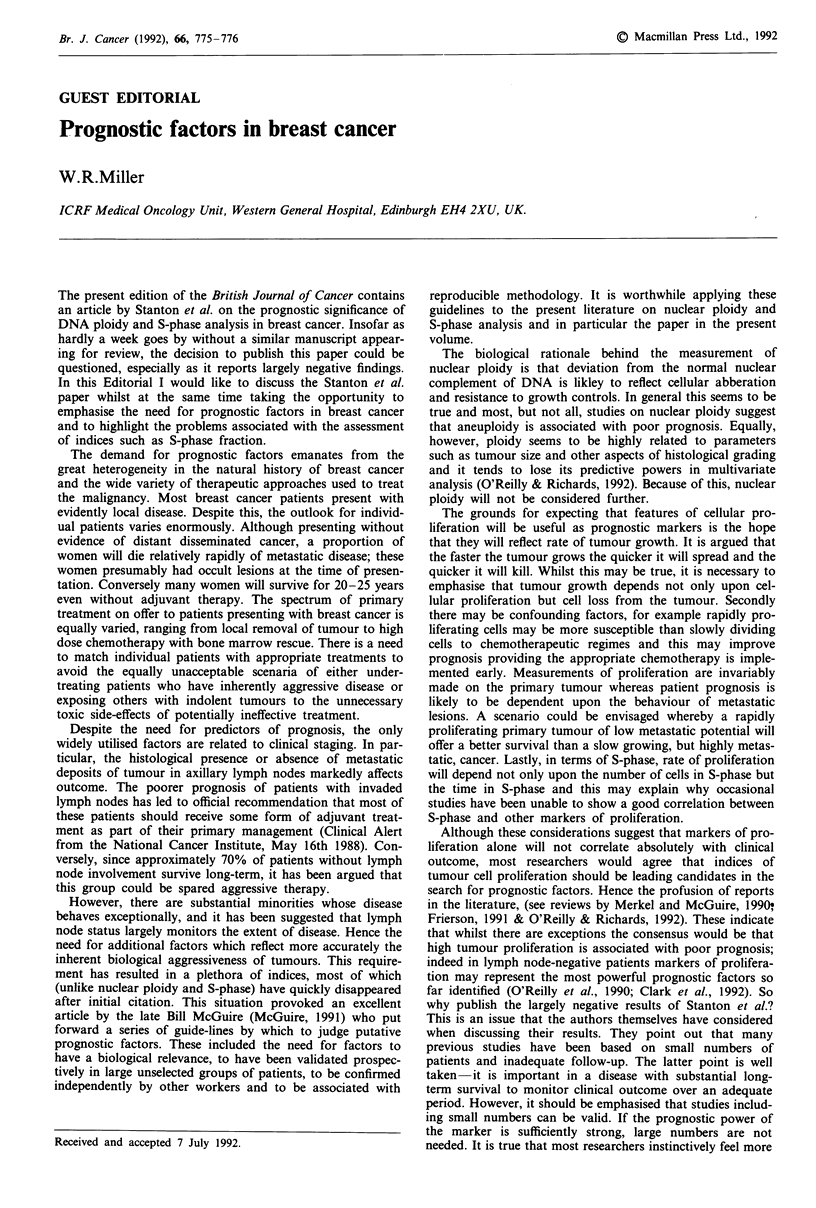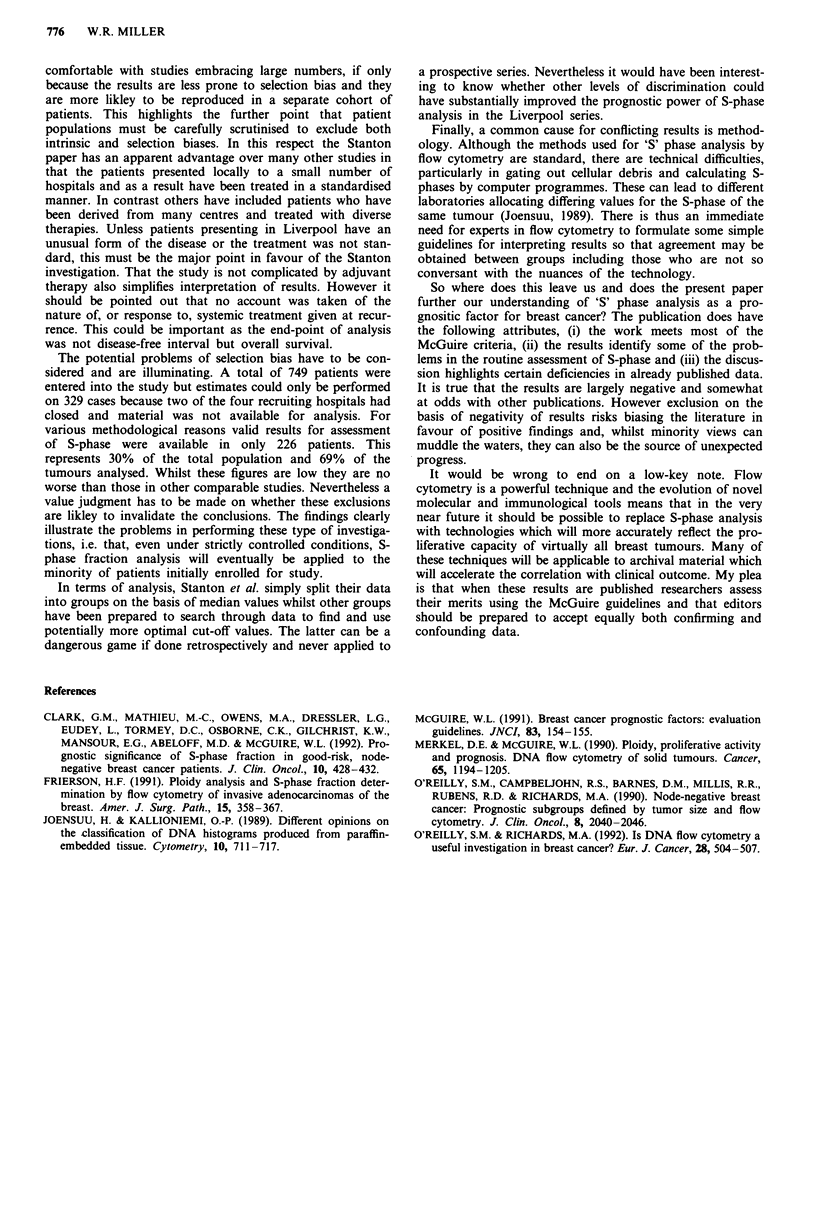# Prognostic factors in breast cancer.

**DOI:** 10.1038/bjc.1992.358

**Published:** 1992-11

**Authors:** W. R. Miller


Br. J. Cancer (1992), 66, 775-776 © Macmillan Press Ltd., 1992

GUEST EDITORIAL

### Prognostic factors in breast cancer

W.R.Miller

*ICRF Medical Oncology Unit, Western General Hospital, Edinburgh EH4 2XU, UK.*

The present edition of the *British Journal of Cancer* contains an article by Stanton *et al.* on the prognostic significance of DNA ploidy and S-phase analysis in breast cancer. Insofar as hardly a week goes by without a similar manuscript appearing for review, the decision to publish this paper could be questioned, especially as it reports largely negative findings. In this Editorial I would like to discuss the Stanton *et al.* paper whilst at the same time taking the opportunity to emphasise the need for prognostic factors in breast cancer and to highlight the problems associated with the assessment of indices such as S-phase fraction.

The demand for prognostic factors emanates from the great heterogeneity in the natural history of breast cancer and the wide variety of therapeutic approaches used to treat the malignancy. Most breast cancer patients present with evidently local disease. Despite this, the outlook for individual patients varies enormously. Although presenting without evidence of distant disseminated cancer, a proportion of women will die relatively rapidly of metastatic disease; these women presumably had occult lesions at the time of presentation. Conversely many women will survive for 20-25 years even without adjuvant therapy. The spectrum of primary treatment on offer to patients presenting with breast cancer is equally varied, ranging from local removal of tumour to high dose chemotherapy with bone marrow rescue. There is a need to match individual patients with appropriate treatments to avoid the equally unacceptable scenaria of either under-treating patients who have inherently aggressive disease or exposing others with indolent tumours to the unnecessary toxic side-effects of potentially ineffective treatment.

Despite the need for predictors of prognosis, the only widely utilised factors are related to clinical staging. In particular, the histological presence or absence of metastatic deposits of tumour in axillary lymph nodes markedly affects outcome. The poorer prognosis of patients with invaded lymph nodes has led to official recommendation that most of these patients should receive some form of adjuvant treatment as part of their primary management (Clinical Alert from the National Cancer Institute, May 16th 1988). Conversely, since approximately 70% of patients without lymph node involvement survive long-term, it has been argued that this group could be spared aggressive therapy.

However, there are substantial minorities whose disease behaves exceptionally, and it has been suggested that lymph node status largely monitors the extent of disease. Hence the need for additional factors which reflect more accurately the inherent biological aggressiveness of tumours. This requirement has resulted in a plethora of indices, most of which (unlike nuclear ploidy and S-phase) have quickly disappeared after initial citation. This situation provoked an excellent article by the late Bill McGuire (McGuire, 1991) who put forward a series of guide-lines by which to judge putative prognostic factors. These included the need for factors to have a biological relevance, to have been validated prospectively in large unselected groups of patients, to be confirmed independently by other workers and to be associated with reproducible methodology. It is worthwhile applying these guidelines to the present literature on nuclear ploidy and S-phase analysis and in particular the paper in the present volume.

The biological rationale behind the measurement of nuclear ploidy is that deviation from the normal nuclear complement of DNA is likley to reflect cellular abberation and resistance to growth controls. In general this seems to be true and most, but not all, studies on nuclear ploidy suggest that aneuploidy is associated with poor prognosis. Equally, however, ploidy seems to be highly related to parameters such as tumour size and other aspects of histological grading and it tends to lose its predictive powers in multivariate analysis (O'Reilly & Richards, 1992). Because of this, nuclear ploidy will not be considered further.

The grounds for expecting that features of cellular proliferation will be useful as prognostic markers is the hope that they will reflect rate of tumour growth. It is argued that the faster the tumour grows the quicker it will spread and the quicker it will kill. Whilst this may be true, it is necessary to emphasise that tumour growth depends not only upon cellular proliferation but cell loss from the tumour. Secondly there may be confounding factors, for example rapidly proliferating cells may be more susceptible than slowly dividing cells to chemotherapeutic regimes and this may improve prognosis providing the appropriate chemotherapy is implemented early. Measurements of proliferation are invariably made on the primary tumour whereas patient prognosis is likely to be dependent upon the behaviour of metastatic lesions. A scenario could be envisaged whereby a rapidly proliferating primary tumour of low metastatic potential will offer a better survival than a slow growing, but highly metastatic, cancer. Lastly, in terms of S-phase, rate of proliferation will depend not only upon the number of cells in S-phase but the time in S-phase and this may explain why occasional studies have been unable to show a good correlation between S-phase and other markers of proliferation.

Although these considerations suggest that markers of proliferation alone will not correlate absolutely with clinical outcome, most researchers would agree that indices of tumour cell proliferation should be leading candidates in the search for prognostic factors. Hence the profusion of reports in the literature, (see reviews by Merkel and McGuire, 1990; Frierson, 1991 & O'Reilly & Richards, 1992). These indicate that whilst there are exceptions the consensus would be that high tumour proliferation is associated with poor prognosis; indeed in lymph node-negative patients markers of proliferation may represent the most powerful prognostic factors so far identified (O'Reilly *et al.*, 1990; Clark *et al.*, 1992). So why publish the largely negative results of Stanton *et al.*? This is an issue that the authors themselves have considered when discussing their results. They point out that many previous studies have been based on small numbers of patients and inadequate follow-up. The latter point is well taken—it is important in a disease with substantial long-term survival to monitor clinical outcome over an adequate period. However, it should be emphasised that studies including small numbers can be valid. If the prognostic power of the marker is sufficiently strong, large numbers are not needed. It is true that most researchers instinctively feel more

Received and accepted 7 July 1992.

comfortable with studies embracing large numbers, if only because the results are less prone to selection bias and they are more likley to be reproduced in a separate cohort of patients. This highlights the further point that patient populations must be carefully scrutinised to exclude both intrinsic and selection biases. In this respect the Stanton paper has an apparent advantage over many other studies in that the patients presented locally to a small number of hospitals and as a result have been treated in a standardised manner. In contrast others have included patients who have been derived from many centres and treated with diverse therapies. Unless patients presenting in Liverpool have an unusual form of the disease or the treatment was not standard, this must be the major point in favour of the Stanton investigation. That the study is not complicated by adjuvant therapy also simplifies interpretation of results. However it should be pointed out that no account was taken of the nature of, or response to, systemic treatment given at recurrence. This could be important as the end-point of analysis was not disease-free interval but overall survival.

The potential problems of selection bias have to be considered and are illuminating. A total of 749 patients were entered into the study but estimates could only be performed on 329 cases because two of the four recruiting hospitals had closed and material was not available for analysis. For various methodological reasons valid results for assessment of S-phase were available in only 226 patients. This represents 30% of the total population and 69% of the tumours analysed. Whilst these figures are low they are no worse than those in other comparable studies. Nevertheless a value judgment has to be made on whether these exclusions are likley to invalidate the conclusions. The findings clearly illustrate the problems in performing these type of investigations, i.e. that, even under strictly controlled conditions, S-phase fraction analysis will eventually be applied to the minority of patients initially enrolled for study.

In terms of analysis, Stanton *et al.* simply split their data into groups on the basis of median values whilst other groups have been prepared to search through data to find and use potentially more optimal cut-off values. The latter can be a dangerous game if done retrospectively and never applied to a prospective series. Nevertheless it would have been interesting to know whether other levels of discrimination could have substantially improved the prognostic power of S-phase analysis in the Liverpool series.

Finally, a common cause for conflicting results is methodology. Although the methods used for 'S' phase analysis by flow cytometry are standard, there are technical difficulties, particularly in gating out cellular debris and calculating S-phases by computer programmes. These can lead to different laboratories allocating differing values for the S-phase of the same tumour (Joensuu, 1989). There is thus an immediate need for experts in flow cytometry to formulate some simple guidelines for interpreting results so that agreement may be obtained between groups including those who are not so conversant with the nuances of the technology.

So where does this leave us and does the present paper further our understanding of 'S' phase analysis as a prognositic factor for breast cancer? The publication does have the following attributes, (i) the work meets most of the McGuire criteria, (ii) the results identify some of the problems in the routine assessment of S-phase and (iii) the discussion highlights certain deficiencies in already published data. It is true that the results are largely negative and somewhat at odds with other publications. However exclusion on the basis of negativity of results risks biasing the literature in favour of positive findings and, whilst minority views can muddle the waters, they can also be the source of unexpected progress.

It would be wrong to end on a low-key note. Flow cytometry is a powerful technique and the evolution of novel molecular and immunological tools means that in the very near future it should be possible to replace S-phase analysis with technologies which will more accurately reflect the proliferative capacity of virtually all breast tumours. Many of these techniques will be applicable to archival material which will accelerate the correlation with clinical outcome. My plea is that when these results are published researchers assess their merits using the McGuire guidelines and that editors should be prepared to accept equally both confirming and confounding data.

### References

CLARK, G.M., MATHIEU, M.-C., OWENS, M.A., DRESSLER, L.G., EUDEY, L., TORMEY, D.C., OSBORNE, C.K., GILCHRIST, K.W., MANSOUR, E.G., ABELOFF, M.D. & MCGUIRE, W.L. (1992). Prognostic significance of S-phase fraction in good-risk, node-negative breast cancer patients. *J. Clin. Oncol.*, **10**, 428-432.

FRIERSON, H.F. (1991). Ploidy analysis and S-phase fraction determination by flow cytometry of invasive adenocarcinomas of the breast. *Amer. J. Surg. Path.*, **15**, 358-367.

JOENSUU, H. & KALLIONIEMI, O.-P. (1989). Different opinions on the classification of DNA histograms produced from paraffin-embedded tissue. *Cytometry*, **10**, 711-717.

MCGUIRE, W.L. (1991). Breast cancer prognostic factors: evaluation guidelines. *JNCI*, **83**, 154-155.

MERKEL, D.E. & MCGUIRE, W.L. (1990). Ploidy, proliferative activity and prognosis. DNA flow cytometry of solid tumours. *Cancer*, **65**, 1194-1205.

O'REILLY, S.M., CAMPBELJOHN, R.S., BARNES, D.M., MILLIS, R.R., RUBENS, R.D. & RICHARDS, M.A. (1990). Node-negative breast cancer: Prognostic subgroups defined by tumor size and flow cytometry. *J. Clin. Oncol.*, **8**, 2040-2046.

O'REILLY, S.M. & RICHARDS, M.A. (1992). Is DNA flow cytometry a useful investigation in breast cancer? *Eur. J. Cancer*, **28**, 504-507.